# Dengue Type 3 Virus, Saint Martin, 2003–2004

**DOI:** 10.3201/eid1105.040959

**Published:** 2005-05

**Authors:** Christophe N. Peyrefitte, Boris A.M. Pastorino, Maël Bessaud, Patrick Gravier, Fabienne Tock, Patricia Couissinier-Paris, Jenny Martial, Patricia Huc-Anais, Raymond Césaire, Marc Grandadam, Hugues J. Tolou

**Affiliations:** * Institut de Médecine Tropicale du Service de Santé des Armées, Marseille Armées, France;; † Université de la Méditerranée, Marseille, France;; ‡ Centre Hospitalier Universitaire de Fort de France, Martinique, France;; § Laboratoire d'Analyse Médicale Lepers, Marigot, Saint Martin, France

**Keywords:** Dengue Virus, French west indies, molecular epidemiology

## Abstract

We describe the spread of a dengue virus during an outbreak in Saint Martin island (French West Indies) during winter 2003–2004. Dengue type 3 viruses were isolated from 6 patients exhibiting clinical symptoms. This serotype had not been detected on the island during the preceding 3 years. Genome sequence determinations and analyses showed a common origin with dengue type 3 viruses isolated in Martinique 2 years earlier.

Dengue virus (DENV) infection is recognized as a major public health problem; >50 million persons are infected each year worldwide (*1*), and the incidence of severe, sometimes lethal, forms of the disease is increasing (*2*). Dengue viruses are mosquitoborne flaviviruses with a single-stranded, nonsegmented, positive-sense RNA genome ≈11 kb in length. Four antigenically distinct serotypes, DENV types 1 to 4, exist (*3*). Infection with any serotype can lead to disease, ranging from mild infection, dengue fever (a generally mild disease with complete recovery), to severe forms (dengue hemorrhagic fever and dengue shock syndrome). Molecular epidemiologic studies have investigated the possibility of a link between particular DENV genotypes or clusters and particular clinical forms of disease (*4**,**5*). Consequently, finding new viral genotypes in areas where they had been absent could be of epidemiologic and clinical interest. A recent work described the emergence and the global spread of DENV-3 subtype III (*5*). Originating from the Sri Lankan/Indian subcontinent, the new variant likely spread to eastern Africa in the 1980s, then to Latin America in the mid-1990s (*5*). Previous work in our laboratory identified a DENV-3 subtype 3 of the same origin in Martinique in 1999 (*6*).

Whereas other Caribbean islands had annual dengue epidemics, the last outbreak reported in Saint Martin (French West Indies) (Figure 1) was in 1977 (*7*). Only DENV-1 was isolated during this epidemic, while DENV-1, DENV-2, and DENV-3 circulated in Puerto Rico at the same time (*8*). In 2000, during an interepidemic period, 1 isolate of DENV-3 was reported in Saint Martin (http://www.carec.org/annrep00/index.html), but to our knowledge its sequence was not determined. During the last 3 years, no further DENV isolates have been reported on the island. However, in December 2003, an outbreak of febrile illness was reported after heavy rains. An estimated 108 persons were infected, and DENV was suspected to be the causative agent by Saint Martin Lepers Laboratory (*9*). During this period, blood samples collected at early and late stages of infections were examined at the Tropical Medicine Institute of the French Armed Forces Medical Service (Institut de Médecine Tropicale du Service de Santé des Armées [IMTSSA]) tropical virology laboratory in Marseille to identify the etiologic agent. DENV-3 was isolated on C6/36 cells (*Aedes albopictus*). Partial genomic sequences were determined for each isolated virus to evaluate the origin and diversity of its spread. This approach should help clarify the geographic distribution of isolates in the Caribbean islands as well as the virus' circulation in and transmission to humans.

**Figure 1:**
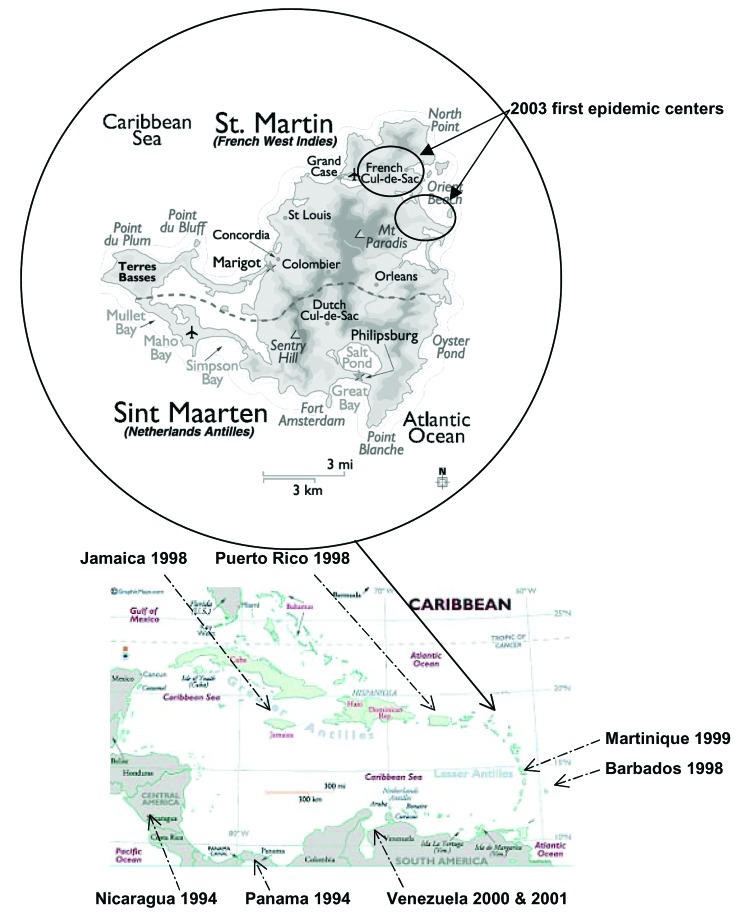
Map of Saint Martin, an island in the Caribbean. The lower map represents the Caribbean subregion. The year of dengue virus type 3 subtype III detection is indicated under the concerned island or country. The upper map is a close-up of Saint Martin. Source: Centre de documentation de l'Institut de Médecine Tropicale du Service de Santé des Armées.

## Materials and Methods

### The Outbreak

**Figure 2:**
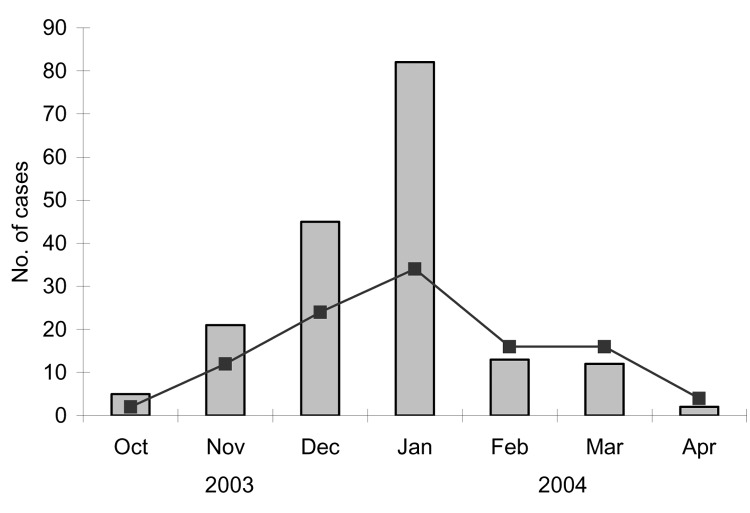
Temporal distribution of the outbreak (adapted from [10]). Bars indicate the suspected dengue patients observed by sentinel physicians; dark squares (curve) indicate laboratory-confirmed cases.

In December 2003, an outbreak of febrile illness was reported (*9*) after heavy rainfalls in Saint Martin (29,000 inhabitants in the French part and 36,000 in the Dutch part). The outbreak seemed to start first in the northeastern part of the island ("Baie orientale" and "Cul de sac" neighborhoods), then spread through the island (Figure 1). The temporal distribution of the outbreak is shown in Figure 2 (*10*). We used a commercial enzyme-linked immunosorbent assay (ELISA) (Eurobio, Courtaboeuf, France) in Saint Martin Lepers Laboratory to determine that, from October 2003 to April 2004, a total of 108 persons were infected; a DENV was suspected as the etiologic agent (*10*). Twelve persons manifesting thrombocytopenia without hemorrhagic signs were hospitalized (*9*).

### Collection and Transport of Samples

Sera from 26 patients with dengue-like syndromes were collected at a local laboratory and transported (by air, at 4°C) to the IMTSSA laboratory in Marseille for serologic diagnosis and etiologic agent identification. All these samples were collected from December 2003 to January 2004, the peak of the outbreak.

### Serologic Diagnosis

In-house immunoglobulin (Ig) M-capture enzyme immunoassays (MAC-ELISA) and IgG sandwich ELISA were used to detect serum IgM and IgG antibodies to Toscana virus, dengue viruses, West Nile virus, and St. Louis encephalitis virus. Briefly, IgM antibodies were captured with rabbit anti-human IgM antibodies (Interchim, Montluçon, France). Toscana, dengue, West Nile, and Saint Louis antigens, prepared on Vero cells and inactivated by betapropiolactone (Sigma-Aldrich, St Quentin Fallavier, France), were added. Specific binding was demonstrated by using a Toscana virus, dengue viruses, West Nile virus, and St. Louis encephalitis virus mouse hyperimmune ascitic fluid virus (*11*) and a goat anti-mouse peroxidase-labeled conjugate (Interchim). For IgG detection, antibodies to Toscana virus, dengue viruses, West Nile virus, and St. Louis encephalitis virus were captured by goat anti-mouse IgG antibodies; viral antigens were followed by test sera; and specific binding was demonstrated by using a peroxidase-labeled goat anti-human IgG conjugate. Serum samples were considered positive if the optical density at 450 nm was >3-fold the mean of negative sera with an OPTImax spectrophotometer (Molecular Devices, Saint Gregoire, France).

### Virus Isolation and Propagation

Work with the infectious virus was carried out in a biosafety level 3 laboratory. Virus isolation was attempted when the first sample could be collected <5 days after onset of illness and when enough serum was collected for serologic analysis (*12*). DENV-3 strains (Table A1) were all isolated from leukocytes of patients. Leukocytes of the samples were directly coincubated with C6/36 cells (*Ae. albopictus*) grown at 28°C in Leibowitz's L15 medium (BioWhittaker Europe, Verniers, Belgium) supplemented with 1% L-glutamine and 2% tryptose phosphate broth. Fetal bovine serum (5% final) was added 1 h later. Supernatants were collected on day 5 after infection.

Indirect immunofluorescence (IF) was performed on C6/36 cells with DENV-1, -2, -3, and -4 serotype–specific monoclonal antibodies (kindly provided by Nick Karabatsos, Centers for Disease Control and Prevention, Fort Collins, Colorado); on mouse hyperimmune ascitic fluids for West Nile virus and Bunyamwera virus; and on grouping fluids for alphaviruses, phleboviruses, and California serogroup viruses. After isolation attempts, remaining sera were used for serologic studies but not for DENV-3–specific reverse transcription–polymerase chain reaction (RT-PCR) because too little serum was available after serologic tests.

### RNA Preparation and cDNA Synthesis

Viral RNA was extracted from 200-μL aliquots of infected cells supernatants by using the High Pure Viral RNA kit (Roche Diagnostics, Meylan, France) following manufacturer's protocol. Two overlapping viral cDNA fragments were generated by RT with Superscript II reverse transcriptase (Invitrogen/Life Technologies, Cergy Pontoise, France) according to the manufacturer protocols with D3/C/863/18 and D3/C/1419/18 primers (Table). Specific primers (Table) designed from the nucleotide sequence of the reference DENV-3 H87 (GenBank accession no. L11423) (*13*) were used for PCR amplification by using AmpliTaq DNA Gold (Applera, Courtaboeuf, France).

### DNA Sequencing

PCR products were purified from 1.5 % agarose gels, by using the QIAquick gel extraction kit (Qiagen, Courtaboeuf, France) and directly sequenced with the direct and reverse primers (Table) and the Big Dye Sequencing kit (Applera). Sequencing was carried out with an automatic sequence analyzer (ABI PRISM 3100, Applera) following the manufacturer's protocol.

Sequences of the Saint Martin DENV isolates were compared to GenBank database DENV sequences (Table A1). Alignments of nucleotide and amino acid sequences of the prM/M and partial E nucleotide sequences (nucleotide numbers 437–1144 by reference to the sequence of the D3H87 strain) were performed with ClustalW1.7 software (*14*). Phylograms were constructed with the MEGA 2 program (*15*), and tree drawing used the Jukes-Cantor algorithm for genetic distance determination and the Neighbor Joining method. The robustness of the resulting tree was tested by 1,000 bootstrap replications.

## Results and Discussion

From October 2003 to April 2004, DENV-3 circulated on the island, and 108 cases of dengue fever were serologically or virologically confirmed in both the Lepers Laboratory and the IMTSSA laboratory, and 12 persons were hospitalized (*10*). If one takes into account the 180 suspected cases observed during this period, the incidence of dengue fever during the epidemic was ≈0.62% (180/29,000) on the French part of the island; this value was consistent with values already observed in the Caribbean region (http://www.carec.org/data/dengue/1998/table2.html). No data were available from the Dutch half.

During this period, an additional 26 blood samples from patients with dengue-like syndromes at early stage of the infection were received in IMTSSA Tropical Virology Laboratory in Marseille. Among them, 13 were positive for dengue infection by presence of IgM, elevation (4-fold) of specific IgG, or both. Virus was isolated from 6 other patients, and DENV-3 was identified by IF using serotype-specific monoclonal antibodies. No fluorescence was observed when DENV-1, DENV-2, or DENV-4 monoclonal antibodies were used. Moreover, IF tests were negative when antibody to West Nile virus, Toscana virus, Bunyamwera virus, alphaviruses, phleboviruses and California serogroup viruses was used. These cases were considered confirmed dengue fever; all the patients recovered fully.

DENV-3 was genetically identified in the 6 IF positive isolates (D3StMart1–D3StMart6, referred to as GenBank accession no. AY750713–AY750718) by using BLAST-NCBI software. Comparison of partial sequences showed a high degree of identity between the strains isolated from patients on Saint Martin: paired identity at the nucleotide level ranged from 99.3% to 100%. When compared to 00PuertoR1 strain of the Latin American cluster defined by Messer (*5*), the values were 99.5%–99.6%. Nucleotide comparison gave the following values: 98.3%–98.4% for East African cluster (*5*), 98.8%–98.9% for Group B (*5*), and 96.8%–96.9% for Group A (*5*). This comparison indicated a close relationship between Saint Martin and Latin American isolates.

The strains from Saint Martin shared an 11-nucleotide insertion between position 10,275 and position 10,276 in the 3´UTR (AGTGAAAAAGA). The same insertion was found in isolates from Martinique 2 years earlier (*6*) and from DENV-3 Sri Lanka (*6*), indicating a probable common ancestor. This finding was consistent with a Sri Lankan origin for DENV-3 subtype III circulating in the Latin American and Caribbean regions (*5*) and with extension of the virus from a single importation event.

**Figure 3:**
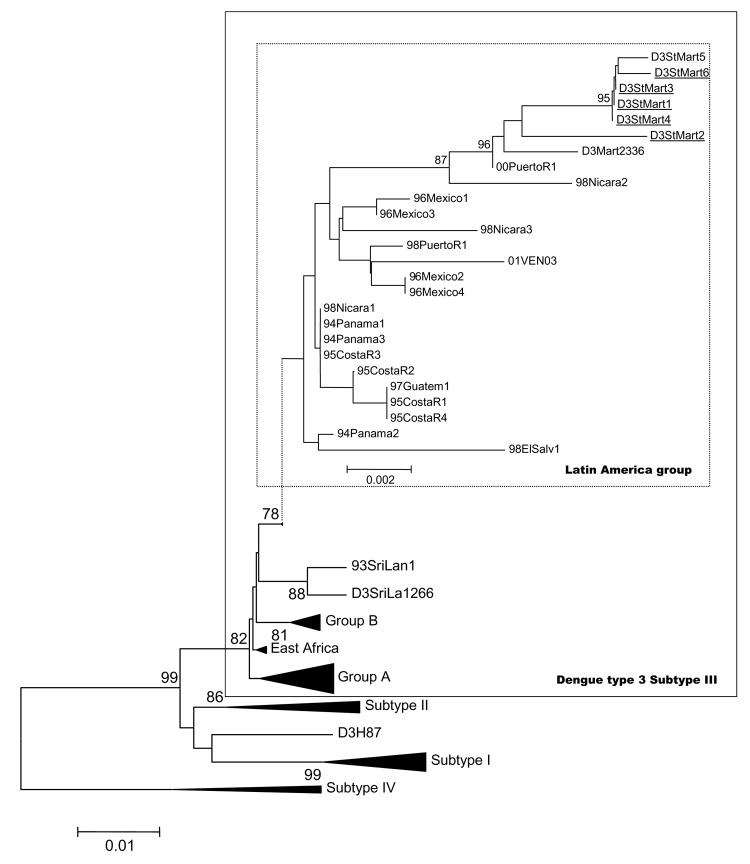
Phylogenetic tree of dengue type 3 subtype III viruses, based on prM/M and partial E nucleotide sequences (nucleotide numbers 437 to 1144) available in GenBank database. Phylograms were constructed with the MEGA 2 program (15), using the Jukes-Cantor algorithm and the neighbor joining method. The percentage of successful bootstrap replicates (1,000 bootstrap replications, confidence probability >90%) is indicated at nodes. The length of branches is proportional to the number of nucleotide changes (percentage of divergence). The strains sequenced in this work are underlined. Dark triangles correspond to viruses of the same group clustering together; dots indicate a change in scale.

A phylogenetic tree was constructed based on partial sequences of the prM/M-E gene region (position 437–1144) of the genomes, including the strains from Saint Martin and several other previously characterized strains from different origins (*16**–**19*). DENV isolated from Saint Martin was grouped in subtype III, as defined by Lanciotti (*17*) (data not shown). Another phylogenetic tree was constructed with the strains analyzed by Messer (*5*) (Figure 3). Despite the high overall similarity of the subtype III sequences, distinct groups could be distinguished. The 6 isolates from Saint Martin clustered together. The small lineage difference of D3StMart2 is supported by a 95-bootstrap value, but no evidence suggests that such difference implies multiple introductions or a longer transmission period. However, Saint Martin DENV-3 fell in the Latin American cluster defined by Messer (*5*), which also includes isolates from Guatemala, Nicaragua, and Mexico. Isolates from Saint Martin were particularly close to Martinique isolates (*6*) and the 00PuertoR1 isolate, which were the geographically and temporally closest isolates of the cluster. The analysis of the deduced amino acid sequences generated a similar phylogram. Altogether, these results indicate that the Saint Martin viral isolate belongs to the genotype Puerto Rico 2000 previously reported (*5*). These results also confirm a common origin for all DENV-3 circulating in the Caribbean and Latin American region; their ancestor probably originated from Sri Lanka. Additional American DENV-3 strain sequences, encompassing the insertion site, should help to confirm this hypothesis.

The question arose whether the introduction from Sri Lanka was a single event in the Latin American region, first appearing in Panama (*5*), then spreading through the Caribbean subregion and South America. In fact, viruses belonging to subtype III were first reported in Nicaragua and Panama in 1994 (*20*). They were then identified in Guatemala from 1996 to 1998; in Puerto Rico, Barbados, and Jamaica in 1998 (*18*); and in Martinique in 1999 (*6*). The introduction of DENV-3 in Saint Martin was reported in 2000 (http://www.carec.org/annrep00/index.html), but the subtype was not determined. No virus isolation was reported for 3 years, until the viruses we characterized were isolated. The rapid extension of DENV-3 belonging to subtype III, which have almost completely replaced preexisting DENV of serotype 1 and 2, is indicative that these particular viruses have adapted to Caribbean and Latin American conditions. Together with previous studies (*5**,**6*), identification of viruses belonging to subtype III in another area of the Caribbean region confirms the remarkable epidemiologic success of this particular lineage of dengue viruses.

Comparable substitution of local DENV by a new genotype was previously described in the Caribbean region (*12*). New viruses, often originating from Southeast Asia, arise as successive waves, replacing previous ones. In this regard, sampling of DENV-3 subtype III virus nucleotide sequences from all countries in the Caribbean and the peri-Caribbean area need to be expanded so that we can understand DENV circulation in the region.

## Appendix

**Table A1:** DENV-3 sequences and corresponding GenBank accession numbers*

Name	Location	Source	Date	Accession no.	Subtype	Group
D3StMart1	Saint Martin island	This study	Dec. 2003	AY750713	III	Latin America
D3StMart2	Saint Martin island	This study	Dec. 2003	AY750714	III	Latin America
D3StMart3	Saint Martin island	This study	Jan. 2004	AY750715	III	Latin America
D3StMart4	Saint Martin island	This study	Jan. 2004	AY750716	III	Latin America
D3StMart5	Saint Martin island	This study	Jan. 2004	AY750717	III	Latin America
D3StMart6	Saint Martin island	This study	Jan. 2004	AY750718	III	Latin America
D3Mart2336	Martinique island	GenBank	Jan. 2001	AY099342	III	Latin America
98Nicara1	Nicaragua	GenBank	1998	AF547245	III	Latin America
98Nicara2	Nicaragua	GenBank	1998	AF547246	III	Latin America
98Nicara3	Nicaragua	GenBank	1998	AF547262	III	Latin America
94Panama1	Panama	GenBank	1994	AF547247	III	Latin America
94Panama2	Panama	GenBank	1994	AF547248	III	Latin America
94Panama3	Panama	GenBank	1994	AF547249	III	Latin America
96Mexico1	Mexico	GenBank	1996	AF547250	III	Latin America
96Mexico2	Mexico	GenBank	1996	AF547251	III	Latin America
96Mexico3	Mexico	GenBank	1996	AF547252	III	Latin America
96Mexico4	Mexico	GenBank	1996	AF547253	III	Latin America
95CostaR1	Costa Rica	GenBank	1995	AF547254	III	Latin America
95CostaR2	Costa Rica	GenBank	1995	AF547255	III	Latin America
95CostaR3	Costa Rica	GenBank	1995	AF547256	III	Latin America
95CostaR4	Costa Rica	GenBank	1995	AF547257	III	Latin America
97Guatem1	Guatemala	GenBank	1997	AF547263	III	Latin America
98PuertoR1	Puerto Rico	GenBank	1998	AF547258	III	Latin America
00PuertoR1	Puerto Rico	GenBank	2000	AF547264	III	Latin America
98ElSalv1	El Salvador	GenBank	1998	AF547259	III	Latin America
01VEN03	Venezuela	GenBank	2001	AF547261	III	Latin America
93SriLan1	Sri Lanka	GenBank	1993	AF547234	III	unclassified
D3SriLa1266	Sri Lanka	GenBank	2000	AY099336	III	unclassified
83SriLan1	Sri Lanka	GenBank	1983	AF547225	III	Group A
83SriLan2	Sri Lanka	GenBank	1983	AF547226	III	Group A
83SriLan3	Sri Lanka	GenBank	1983	AF547227	III	Group A
83SriLan4	Sri Lanka	GenBank	1983	AF547228	III	Group A
84SriLan1	Sri Lanka	GenBank	1984	AF547229	III	Group A
85SriLan	Sri Lanka	GenBank	1985	AF547241	III	Group A
89SriLan2	Sri Lanka	GenBank	1989	AF547231	III	Group A
89SriLan1	Sri Lanka	GenBank	1989	AF547230	III	Group B
89SriLan3	Sri Lanka	GenBank	1989	AF547232	III	Group B
90SriLan1	Sri Lanka	GenBank	1990	AF547233	III	Group B
94SriLan1	Sri Lanka	GenBank	1994	AF547235	III	Group B
97SriLan1	Sri Lanka	GenBank	1997	AF547242	III	Group B
81-91SriLanA	Sri Lanka	GenBank	1981,1991	L11431,L11436-L11438	III	Group B
85Mozamb1	Mozambique	GenBank	1985	AF547236	III	East Africa
85Mozamb2	Mozambique	GenBank	1985	AF547237	III	East Africa
85Mozamb3	Mozambique	GenBank	1985	AF547238	III	East Africa
91Kenya	Kenya	GenBank	1991	AF547239	III	East Africa
93Somalia	Somalia	GenBank	1993	AF547240	III	East Africa
73-86Thailand	Thailand	GenBank	1973, 1986	L11441-L11442,L11620	II	reference
83Philipp	Philippines	GenBank	1983	L11432	I	reference
89Tahiti	Tahiti	GenBank	1989	L111619	I	reference
92Fiji	Fiji	GenBank	1992	L11422	I	reference
73-85Indones	Indonesia	GenBank	1973, 1985	L11425,L11426,L11428	I	reference
74,81Malaysi	Malaysia	GenBank	1974, 1981	L11429, L11427	I	reference
D3H-87	Philippines	GenBank	1956	L11423	I	reference
63,77PuertoR	Puerto Rico	GenBank	1963,77	L11433, L11434	IV	reference
65Tahiti	Tahiti	GenBank	1965	L11439	IV	reference

**Table:** Primers used for DENV-3 genome amplification and sequencing*

Primer name	(5´→ 3´) Sequence	Genome position†
D3/D/1/17	AGTTGTTAGTCTACGTG	1–17
D3/D/740/18	GGACTGGACACACGCACT	740–757
D3/D/9970/18	GTGGATGACTACAGAAGA	9970–9987
D3/C/863/18	AATGGGCAAGAAATAGGG	863–846
D3/C/1419/18	ATCTCAGCCGTAACTCCC	1419–1402
D3/C/10696/18	AGAACCTGTTGATTCAAC	10696–10679

*DENV, dengue virus. †According to the DENV-3 H87 reference sequence.
